# High risk of burnout in medical students in Serbia, by gender: A cross-sectional study

**DOI:** 10.1371/journal.pone.0256446

**Published:** 2021-08-20

**Authors:** Irena Ilic, Ivana Zivanovic Macuzic, Sanja Kocic, Milena Ilic

**Affiliations:** 1 Faculty of Medicine, University of Belgrade, Belgrade, Serbia; 2 Department of Anatomy, Faculty of Medical Sciences, University of Kragujevac, Kragujevac, Serbia; 3 Department of Social Medicine, Faculty of Medical Sciences, University of Kragujevac, Kragujevac, Serbia; 4 Department of Epidemiology, Faculty of Medical Sciences, University of Kragujevac, Kragujevac, Serbia; Universidad Miguel Hernandez de Elche, SPAIN

## Abstract

**Introduction:**

Burnout syndrome is common among medical students, but findings about the gender differences in burnout are not consistent. The aim of this study was to assess high risk of burnout syndrome among medical students at one University in Serbia, by gender.

**Method:**

A cross-sectional study was conducted at the Faculty of Medical Sciences, University of Kragujevac in 2014. The Maslach Burnout Inventory—Student Survey was used for assessment of burnout level. A questionnaire on basic socio-demographic characteristics (age, gender, marital status, habits, etc.) and academic performance (year of study, cumulative total average grade, length of study, housing, study financing, etc.) was used. The study included a total of 760 medical students (760/836 medical students; participation rate: 90.9%). Logistic regression analysis was used to determine odds ratios (OR) with 95% confidence intervals (95% CI).

**Results:**

Significant gender differences were detected in prevalence of high risk of burnout syndrome (male students– 19.0% vs. female students– 12.8%, p = 0.024). A significant independent predictor of high risk for burnout syndrome in male medical students was study year (p for trend = 0.011), while in female medical students–study year (p for trend = 0.002) and use of sedatives (adjusted OR = 5.74, 95% CI = 1.96–16.77, p = 0.001).

**Conclusion:**

Our results indicate the need to assess the risk of burnout syndrome at the very beginning of medical studies, in order to more effectively control the modifiable risk factors.

## Introduction

Medical students are continuously exposed to psychosocial stressors during their studies, which, if persistent, can lead to burnout syndrome [1–3]. Student burnout syndrome is primarily related to academic obligations and refers to the feeling of exhaustion due to the demands of studies, a cynical attitude towards studies and the perception of one’s own incompetence as a student [3, 4].

A comprehensive review and meta-analysis showed that in the period 2000–2017 in countries around the world different rates of prevalence of burnout syndrome in medical students were noted, with a range of 7.0% to 75.2% [5]. Recent systematic reviews indicate that at least half of medical students in the developed world have experienced some form of burnout during their studies [6, 7]. The high level of burnout among medical students is the result of the growing degree of curriculum difficulty, and the requirement to absorb a great deal of information over short periods of time, then frequent contact with seriously ill people and death, difficult career choices and financial burdens [8].

Little data is available on burnout syndrome in students from developing countries and it seems that this concept has not been well researched in medical students outside the developed world. Burnout syndrome among medical students has drawn significant scientific attention over the recent years: in 2020 in Cyprus the burnout prevalence was 18.1% [9], approximately 1 in 11 students experienced a high risk of burnout among medical students at Sun Yat-sen University in China [10], while burnout was reported among 54.5% medical students in Uganda [11].

Among the demographic factors associated with burnout in medical students, gender shows conflicting results: in some studies, no statistically significant association was found between burnout prevalence and gender [10, 12, 13], unlike other studies [14–16]. A systematic review of experiences of burnout in medical students in Chinese medical schools reported gender as a significant predictor of burnout, with males experiencing a greater degree of suffering than females [17]. While one meta-analysis showed that male gender was more associated with burnout in medical students in Brazil [18], a recent meta-analysis detected no effect of gender on the prevalence of burnout in medical students [19].

According to the available literature, research dedicated to the identification of predictors of high risk for burnout syndrome in medical students in Serbia is insufficient. The accelerated development of medical sciences indicates the need to assess the frequency of burnout syndrome, as well as the predictors of burnout in medical students. The main goal of this study was to assess the prevalence of high risk for burnout syndrome in medical students in Serbia, to consider differences by gender and to identify factors related to them.

## Methods

### Setting

This study was conducted at the Faculty of Medical Sciences of the University of Kragujevac, Serbia. The Faculty of Medical Sciences has been accredited to organize teaching within the study program of integrated academic studies of medicine for the acquisition of the academic title Doctor of Medicine. Medical education in the Republic of Serbia follows the Bologna Process Principles. To enroll at a Medical Faculty, an entrance exam is mandatory. There are two options concerning students’ fees: self-financing students and budget-financed students. The studies of medicine last 6 years or 12 semesters of training, taking place in the form of: lecture classes, laboratory sessions, other forms of active teaching (seminars, etc), clinical practical instruction (last 3 years). The study programme consists of 35 obligatory and 15 elective courses out of total 50 courses. All courses last one semester. The teaching process is held by the use of interactive programmes oriented toward the students, with constant checking of their knowledge. Methods of lecturing include problem-oriented lectures and small-group work (up to six students). Consequently, since 2010, Faculty of Medical Sciences enrolls a relatively small number of students (about 90), in order to succeed in fulfilling the set standards of the quality of programme. The programme also provides professional practice on several courses (surgery, gynecology, etc.) lasting one week each, during which students continuously spend 24 hours in hospital. Medical faculty uses grades from 5 to 10. All students have to acquire at least 6 (the lowest passing grade). Student assessment is performed by continuous monitoring of a student’s work and on the basis of points gained in fulfilling pre-examination obligations and taking exams.

### Study design

The study was conducted as an epidemiological analytical observational study with cross-sectional study design for estimating the prevalence and level of risk for burnout syndrome, as well as factors associated with burnout syndrome in medical students.

### Study population

The study population consisted of all medical students enrolled in the academic integrated studies at the Faculty of Medical Sciences Kragujevac during the academic study year 2013/2014. The main criterion for inclusion in the study was the attendance of classes at the time of the study. One group of students was visited only once for survey. All students who met the inclusion criteria were eligible to participate in the study. Only those medical students who, before starting the survey, gave voluntary, informed written consent to participate in the research were included in this study.

### Sample size calculation

An a-priori sample size calculation was performed using the Epi Info StatCalc software (Centers for Disease Control and Prevention, Atlanta, Georgia, USA). Based on the population size of 836, acceptable margin of error of 5%, expected frequency of 22.6% [16] and 99.99% confidence level, the minimum sample size was 467. When adjusted for a potential non-response rate of 15%, the minimum sample size was estimated at 538.

Responses to the questionnaire were received from 760 medical students (response rate was 90.9%; 760/836). Responses which were not valid or fully complete were not analyzed.

### Data collection

Data collection was conducted in the auditoriums of the Faculty of Medical Sciences. Before the beginning of the study, ie the survey, a letter/notification was sent to the heads of departments requesting their cooperation in this research, regarding the use of first few minutes of class time in the auditorium to explain the research and administer questionnaires to students. Also, students received written information on the research, which explained the protocol and goals of the study and emphasized the anonymity and voluntary participation of students. During this study, medical students answered the questionnaire in the presence of medical doctors (authors M.I. and I.I.) who were available to address any difficulties in students’ understanding of certain issues. During the whole time, students had the opportunity to ask questions if something was unclear about the research. Completing the questionnaire took about 15 (±5) minutes.

### Instruments

The data on each participant was collected by self-reported questionnaires. The Maslach Burnout Inventory—Student Survey (MBI-SS) [4, 20, 21] and an epidemiological questionnaire were used in this study and administered via paper-pencil format.

Epidemiological questionnaire was designed for the purpose of examining certain sociodemographic (such as age, gender, marital status, children, housing, completed secondary school), academic (study year, study financing, length of study, cumulative total average grade, re-enrollment in the academic year), and lifestyle/health facts (such as cigarettes smoking, alcohol consumption, sports and recreation activity, personal medical history for chronic diseases, use of sedatives).

The MBI-SS questionnaire is an internationally accepted standard for measuring burnout, which Schaufeli and co-authors designed to measure the level of risk for burnout syndrome in students [4, 20, 21]. The MBI-SS questionnaire consists of 15 statements divided into 7 categories ranging from 0 to 6 (0—Never, 1 –A few times a year or less, 2—Once a month or less, 3 –A few times a month, 4—Once a week, 5 –A few times a week, 6 –Every day). These are 15 statements about feelings of students related to the university, ie about feelings due to one’s own academic work. MBI-SS is divided into three "subscales", which independently measure levels of risk for burnout syndrome. The Emotional Exhaustion subscale consists of 5 items, the Cynicism subscale of 4 items and the Academic Efficacy subscale of 6 items. All subscale scores are presented in 3 categories of burnout risk—as low, moderate and high risk of burnout. Three-dimensional criteria (high scores for Emotional Exhaustion and Cynicism, as well as a low score for Academic Efficiency) were used as criteria for confirming burnout.

Our study is the first and, still to-date, the only study that used validated MBI-SS questionnaire in the Serbian language in a sample of medical students [22]. According to the MBI-SS test-authors [3, 4], and as suggested by some other authors [5, 17, 18], prevalence of burnout syndrome was linked to the country-specific factors, cutoff-criteria for burnout syndrome, etc. According to the strong recommendation of the MBI-test authors [3, 4], the subscale scores are based on the 66th percentile of exhaustion and cynicism, and on the 33rd percentile of efficiency. Therefore, in our study, the participants were classified as having the high level of burnout syndrome when their scores were high for emotional exhaustion (score greater than 14) and cynicism (score greater than 6), and low for academic efficiency (score less than 23).

The license for the questionnaire MBI-SS was obtained directly from the current owner, ie from Mind Garden (Menlo Park, CA, USA). The linguistic adaptation and validation of the MBI-SS questionnaire, based on internationally accepted methodology, were performed before the beginning of this research [22]. The Serbian version of the MBI-SS scale [22] applied in this study had good psychometric characteristics, with the reliability expressed by the Cronbach’s alpha coefficient for all domains being very high (Cronbach’s α coefficients for Emotional Exhaustion, Cynicism, and Academic Efficacy were 0.869, 0.856, and 0.852, respectively), while the test-retest reliability showed that the correlation coefficients were at the 0.01 level for all three subscales.

### Statistical analysis

In the analysis of data, Chi-square tests were used to determine differences between male and female medical students in key categorical characteristics. In order to estimate the association between gender and high risk of burnout syndrome, univariate and multivariate logistic regression methods were used to determine odds ratios (OR) with 95% confidence intervals (95%CI). After that, ORs were adjusted for all variables that were related to burnout syndrome in univariate analyses at a p value of < 0.10. Also, adjusting was performed for variables which, according to literature data, were associated to burnout (marital status, children, completed secondary school, study financing, cumulative total average grade, alcohol consumption, sports), although they did not differ significantly between males and females in the present study. Age, smoking status and frequency of alcohol consumption were not included in the multivariate models because of their collinearity with some of the variables. Model fit was assessed by the Hosmer-Lemeshow test of goodness of fit and Cox and Snell’s and Nagelkerke’s Pseudo R square measures. A test for linear trend in risk was based on the logistic regression model.

A value of p < 0.050 was considered significant. All statistical analyses were conducted using the SPSS software (version 20.0, Chicago, IL).

### Ethical consideration

This study is a part of a research approved by the Ethics Committee of the Faculty of Medical Sciences, University of Kragujevac (Ref. No.: 01–1176).

## Results

Compared to males, significantly more females completed secondary medical school before enrolling medical faculty (p = 0.002), had a partner (p = 0.002), had shorter length of studies (p = 0.026), and engaged less in sports and recreational activities (p = 0.000, for both) (Table 1). Cigarette smoking was more common in males than females (37.2% males versus 29.1% females, p = 0.023).

**Table 1:** Socio-demographic characteristics of medical students, by gender.

		Male (N = 269)	Female (N = 491)	
Variables		N (%)	N (%)	p
**Age (years)**				
	≤ 21	67 (24.9)	110 (22.4)	
	22–24	107 (39.8)	220 (44.8)	
	≥ 25	95 (35.3)	161 (32.8)	*0*.*403*
**Study year**				
	1^st^	37 (13.8)	56 (11.4)	
	2^nd^	32 (11.9)	60 (12.2)	
	3^rd^	36 (13.4)	63 (12.8)	
	4^th^	35 (13.0)	103 (21.0)	
	5^th^	71 (26.3)	102 (20.8)	
	6^th^	58 (21.6)	107 (21.8)	*0*.*097*
**Housing**				
	In own home	31 (11.5)	39 (7.9)	
	With parents	91 (33.8)	176 (35.8)	
	As subtenants	127 (47.3)	219 (44.7)	
	In student dormitory	20 (7.4)	57 (11.6)	*0*.*114*
**Study financing**				
	State-sponsored	202 (75.1)	392 (79.8)	
	Self-funded	67 (24.9)	99 (20.2)	*0*.*130*
**Completed secondary school**				
	Grammar school	112 (41.6)	150 (30.5)	
	Medical school	157 (58.4)	341 (69.5)	*0*.*002*
**Marital status**				
	With partner	94 (34.9)	228 (46.4)	
	Without partner	175 (65.1)	263 (53.6)	*0*.*002*
**Length of study (years)**				
	≤ 6	217 (80.7)	426 (86.8)	
	> 6	52 (19.3)	65 (13.2)	*0*.*026*
**Cumulative total average grade**				
	Low	100 (37.2)	176 (35.8)	
	High	169 (62.8)	315 (64.2)	*0*.*716*
**Repeat-year students**				
	No	196 (72.9)	379 (77.2)	
	Yes	73 (27.1)	112 (22.8)	*0*.*184*
**Cigarette smoking**				
	Never	169 (62.8)	348 (70.9)	
	Ever	100 (37.2)	143 (29.1)	*0*.*023*
**Smoking status**				
	Non smokers	169 (62.9)	348 (71.9)	
	Former smokers	48 (17.8)	52 (10.6)	
	Current smokers	52 (19.3)	91 (18.5)	*0*.*013*
**Alcohol consumption**				
	No	67 (24.9)	238 (48.5)	
	Yes	202 (75.1)	253 (51.5)	*0*.*000*
**Frequency of alcohol consumption**				
	Non drinkers	67 (24.9)	238 (48.5)	
	1–2 times a year	15 (5.6)	51 (10.4)	
	1–2 times a month	125 (46.5)	177 (36.0)	
	1–2 times a week	55 (20.4)	22 (4.5)	
	Every day	7 (2.6)	3 (0.6)	*0*.*000*
**Sports**				
	Yes	159 (59.1)	126 (25.7)	
	No	110 (40.9)	365 (74.3)	*0*.*000*
**Recreational activity**				
	Yes	234 (87.0)	347 (70.7)	
	No	35 (13.0)	144 (29.3)	*0*.*000*
**Positive personal medical history**				
	No	259 (96.3)	455 (92.7)	
	Yes	10 (3.7)	36 (7.3)	*0*.*046*
**Use of sedatives**				
	No	262 (97.4)	472 (96.1)	
	Yes	7 (2.6)	19 (3.9)	*0*.*358*

p *(*probability, value according to Chi-square test).

Among medical students, prevalence of high risk of burnout syndrome was significantly higher in males (19.0%) than in females (12.8%), p = 0.024 (Table 2).

**Table 2:** High risk of burnout syndrome in medical students, by gender.

		Burnout syndrome–high risk		
		Absent	Present	
Gender		N (%)	N (%)	p
	**Male**	218 (81.0)	51 (19.0)	
	**Female**	428 (87.2)	63 (12.8)	*0*.*024*
Total		646 (85.0%)	114 (15.0)	

p *(*probability value according to Chi-square test).

Risk for high level of burnout syndrome was significantly increased in both genders in third study year: in females (p = 0.004) with statistical significance for trend (p = 0.005), and in males (p = 0.008) but without statistical significance for trend (p = 0.061) (Table 3). Cigarette smoking was associated with high risk of burnout in female medical students only (p = 0.095). The habit of drinking 1–2 times a week was significantly more common in female medical students who had high risk of burnout syndrome (p = 0.015). Risk of burnout syndrome increased with frequency of alcohol consumption both in males and females (with significance for trend p = 0.076 and p = 0.052, respectively). Use of sedatives was linked to high risk of burnout in female medical students only (p = 0.000).

**Table 3:** Characteristics of medical students with high risk of burnout syndrome, by gender.

		Male (N = 269)				Female (N = 491)			
		Burnout syndrome (high risk)				Burnout syndrome (high risk)			
		Absent	Present			Absent	Present		
Variables		N (%)	N (%)	OR (95% CI)	*p* *	N (%)	N (%)	(95% CI)	*p* *
**Study year**									
	1^st^	34 (15.6)	3 (5.9)	1.00**		53 (12.4)	3 (4.8)	1.00**	
	2^nd^	27 (12.4)	5 (9.8)	2.10 (0.46–9.58)	*0*.*338*	59 (13.8)	1 (1.6)	0.30 (0.03–2.97)	*0*.*303*
	3^rd^	23 (10.6)	13 (25.5)	6.41 (1.64–25.02)	*0*.*008*	46 (10.7)	17 (27.0)	6.53 (1.80–23.70)	*0*.*004*
	4^th^	31 (14.2)	4 (7.8)	1.46 (0.30–7.06)	*0*.*636*	90 (21.0)	13 (20.6)	2.55 (0.70–9.37)	*0*.*158*
	5^th^	56 (25.7)	15 (29.4)	3.04 (0.82–11.26)	*0*.*097*	89 (20.8)	13 (20.6)	2.58 (0.70–9.48)	*0*.*153*
	6^th^	47 (21.6)	11 (21.6)	2.65 (0.69–10.24)	*0*.*157*	91 (21.3)	16 (25.4)	3.11 (0.87–11.16)	*0*.*082*
	***p for trend				*0*.*061*				*0*.*005*
**Completed secondary school**									
	Grammar school	93 (42.7)	19 (37.3)	1.00**		130 (30.4)	20 (31.7)	1.00**	
	Medical school	125 (57.3)	32 (62.7)	1.25 (0.67–2.35)	*0*.*481*	298 (69.6)	43 (68.3)	0.94 (0.53–1.66)	*0*.*825*
**Marital status**									
	With partner	76 (34.9)	18 (35.3)	1.00**		198 (46.3)	30 (47.6)	1.00**	
	Without partner	142 (65.1)	33 (64.7)	0.98 (0.52–1.86)	*0*.*954*	230 (53.7)	33 (52.4)	0.95 (0.56–1.61)	*0*.*840*
**Length of study (years)**									
	≤ 6	179 (82.1)	38 (74.5)	1.00**		375 (87.6)	51 (81.0)	1.00**	
	> 6	39 (17.9)	13 (25.5)	1.57 (0.77–3.22)	*0*.*219*	53 (12.4)	12 (19.0)	1.67 (0.83–3.32)	*0*.*149*
**Cumulative total average grade**									
	Low	76 (34.9)	24 (47.1)	1.00**		150 (35.0)	26 (41.3)	1.00**	
	High	142 (65.1)	27 (52.9)	0.60 (0.33–1.12)	*0*.*109*	278 (65.0)	37 (58.7)	0.77 (0.45–1.32)	*0*.*337*
**Repeat-year students**									
	No	163 (74.8)	33 (64.7)	1.00**		336 (78.5)	43 (68.3)	1.00**	
	Yes	55 (25.2)	18 (35.3)	1.62 (0.84–3.10)	*0*.*148*	92 (21.5)	20 (31.7)	1.70 (0.95–3.03)	*0*.*073*
**Cigarette smoking**									
	Never	138 (63.3)	31 (60.8)	1.00**		309 (72.2)	39 (61.9)	1.00**	
	Ever	80 (36.7)	20 (39.2)	1.11 (0.60–2.08)	*0*.*738*	119 (27.8)	24 (38.1)	1.60 (0.92–2.77)	*0*.*095*
**Smoking status**									
	Non smokers	138 (63.3)	31 (60.8)	1.00**		309 (72.2)	39 (61.9)	1.00**	
	Former smokers	40 (18.3)	8 (15.7)	0.89 (0.38–2.09)	*0*.*790*	42 (9.8)	10 (15.9)	1.89 (0.88–4.06)	*0*.*104*
	Current smokers	40 (18.3)	12 (23.5)	1.34 (0.63–2.84)	*0*.*452*	77 (18.0)	14 (22.2)	1.44 (0.75–2.79)	*0*.*278*
	***p for trend				*0*.*679*				*0*.*202*
**Alcohol consumption**									
	No	52 (23.9)	15 (29.4)	1.00**		209 (48.8)	29 (46.0)	1.00**	
	Yes	166 (76.1)	36 (70.6)	0.75 (0.38–1.48)	*0*.*410*	219 (51.2)	34 (54.0)	1.12 (0.66–1.90)	*0*.*678*
**Frequency of alcohol consumption**									
	Non drinkers	52 (23.9)	15 (29.4)	1.00**		209 (48.8)	29 (46.0)	1.00**	
	1–2 times a year	13 (6.0)	2 (3.9)	0.53 (0.11–2.63)	*0*.*440*	48 (11.2)	3 (4.8)	0.45 (0.13–1.54)	*0*.*204*
	1–2 times a month	109 (50.0)	16 (31.4)	0.51 (0.23–1.11)	*0*.*089*	154 (36.0)	23 (36.5)	1.08 (0.60–1.93)	*0*.*805*
	1–2 times a week	40 (18.3)	15 (29.4)	1.30 (0.57–2.97)	*0*.*534*	15 (3.5)	7 (11.1)	3.36 (1.27–8.94)	*0*.*015*
	Every day	4 (1.8)	3 (5.9)	2.60 (0.52–12.92)	*0*.*243*	2 (0.5)	1 (1.6)	3.60 (0.32–41.00)	*0*.*301*
	***p for trend				*0*.*076*				*0*.*052*
**Sports**									
	Yes	132 (60.6)	27 (52.9)	1.00**		111 (25.9)	15 (23.8)	1.00**	
	No	86 (39.4)	24 (47.1)	1.36 (0.74–2.52)	*0*.*321*	317 (74.1)	48 (76.2)	1.12 (0.60–2.08)	*0*.*719*
**Recreational activity**									
	Yes	188 (86.2)	46 (90.2)	1.00**		309 (72.2)	38 (60.3)	1.00**	
	No	30 (13.8)	5 (9.8)	0.68 (0.25–1.85)	*0*.*452*	119 (27.8)	25 (39.7)	1.71 (0.99–2.95)	*0*.*055*
**Positive personal medical history**									
	No	210 (96.3)	49 (96.1)	1.00**		400 (93.5)	55 (87.3)	1.00**	
	Yes	8 (3.7)	2 (3.9)	1.07 (0.22–5.20)	*0*.*932*	28 (6.5)	8 (12.7)	2.08 (0.90–4.79)	*0*.*086*
**Use of sedatives**									
	No	212 (97.2)	50 (98.0)	1.00**		417 (97.4)	55 (87.3)	1.00**	
	Yes	6 (2.8)	1 (2.0)	0.71 (0.08–6.00)	*0*.*750*	11 (2.6)	8 (12.7)	5.51 (2.13–14.30)	*0*.*000*

* *p*–probability, value according to univariate logistic regression analysis

** Reference category

*** *p* for trend (according to logistic regression).

Abbreviations: OR–Odds Ratio; 95% CI—95% Confidence Interval.

Analysis of high risk of burnout syndrome in medical students by gender revealed that the increase in risk for burnout in both genders was independently associated with study year: in males–p for trend = 0.011 (for third study year: adjusted OR = 8.17, 95%CI = 1.96–33.98, p = 0.004), and in females–p for trend = 0.002 (for third study year: adjusted OR = 8.35, 95%CI = 2.14–32.60, p = 0.002) (Table 4). In female medical students only, high risk of burnout was significantly associated with use of sedatives (adjusted OR = 5.74, 95%CI = 1.96–16.77; p = 0.001).

**Table 4:** High risk of burnout in medical students, by gender: multivariate logistic regression.

Gender	Variables		Adjusted* OR	95% CI	*p*
***Male***					
	**Study year**				
		1^st^	1.00**		
		2^nd^	2.29	0.49–10.80	0.294
		3^rd^	8.17	1.96–33.98	0.004
		4^th^	0.93	0.17–5.13	0.934
		5^th^	2.01	0.50–8.12	0.326
		6^th^	1.57	0.36–6.90	0.552
		*** *p for trend*			*0*.*011*
***Female***					
	**Study year**				
		1^st^	1.00**		
		2^nd^	0.37	0.04–3.79	0.401
		3^rd^	8.35	2.14–32.60	0.002
		4^th^	2.73	0.68–10.98	0.158
		5^th^	2.53	0.62–10.37	0.198
		6^th^	3.69	0.91–14.96	0.067
		*** *p for trend*			*0*.*002*
	**Use of sedatives**				
		No	1.00**		
		Yes	5.74	1.96–16.77	*0*.*001*

* Adjusted for year of study, marital status, children, completed secondary school, study financing, cumulative total average grade, re-enrollment in the academic year, cigarette smoking, alcohol consumption, sports, recreational activity, positive personal medical history, use of sedatives

** *Reference category*

*** *p* for trend (according to logistic regression).

Abbreviations: OR–Odds Ratio; 95% CI—95% Confidence Interval; *p*–probability, value according to multivariate logistic regression analysis.

## Discussion

High risk for burnout syndrome in medical students in our study was noted in 15.0% of participants, significantly more common in males (19.0%) than in females (12.8%). A significant independent predictor of high risk for burnout in medical students of both genders was the year of study. In female medical students, the predictor of high risk for burnout was the use of sedatives.

A recent meta-analysis suggests that one in two medical students worldwide suffers from burnout: the prevalence of burnout (including 17,431 medical students in 2010–2017) was 44.2% (8060 students suffered from burnout) [19]. The prevalence of burnout was higher in countries in Oceania (55.9%) and the Middle East (53.7%) than in countries on other continents (North America—45.8%, Asia—40.6%, Europe—27,5%, South and Central America—26.0%). In the United States, more than half of medical students are affected by burnout during their medical education [6].

A high risk for burnout syndrome in this study was noted in 15.0% of students. Compared to medical students in Kragujevac, a lower prevalence of burnout was observed in medical students of two universities in Brazil (10.3% and 14.9%) [23, 24] and in preclinical medical students in Spain (14.8%) [16], while a higher prevalence was found among medical students in the United Kingdom (26.7%) [25], Ethiopia (34.0%) [26], Pakistan (30.6%) [27], as well as in the United States, India, Malaysia, and Saudi Arabia (45% to 70%) [8, 17, 28–30]. Some of the possible reasons for the differences in the frequency of burnout syndrome among medical students include differences in culture, socioeconomic status, and study population [31]. Also, some studies included only third- and fourth-year medical students, while our study included students of all six years of studies. In addition, different burnout assessment questionnaires and sample sizes may contribute to differences in the prevalence of burnout syndrome.

The results of previous research are not consistent: while some authors have not found a link between gender and burnout in medical students [12–14, 25, 27, 32, 33], other authors have suggested such a link [17]. Maslach considered that gender is not one of the main factors for burnout in employees and that the differences between men and women are very small or non-existent [34]. Santen [12] and Galán [16] did not find a significant association between burnout and gender. On the other hand, studies in Lebanon [35], India [36], and Pakistan [27] found that female gender was significantly associated with burnout in medical students. In contrast, among medical students in the UK [25] and in Brazil [23] a significantly higher prevalence of burnout was observed in males than in females. The reason for the lower risk **o**f burnout in female medical students in our study is not clear. One possible explanation may be that the majority of participants in our study were female medical students (almost 70%), which may have alleviated some of the pressures that women have experienced in previous decades in order to equalize with their peers, to prove themselves in areas dominated by men. Namely, during the previous few decades, female students were in minority and had to prove themselves more and put in more effort, such as more frequent attendance, better learning skills and motivation compared to male students [37]. Some authors have indicated that women are less likely to experience challenging or threatening events as stressful compared to men [38–40], while other studies have noted opposite findings [41]. Also, research suggests that female students often had better social support and showed rational choices in terms of life priorities [38, 42]. Additional studies investigating genetic and hormonal characteristics and their impact on the effect of sex on the risk of burnout syndrome may help to understand this association [43]. Besides, these results may also reflect the existence of confounding / secondary association of gender with some other characteristics [44].

In this study, a significant association was found between the third academic year and high risk for burnout syndrome in both genders, and a significant declining trend in the frequency of high risk was observed with years of study. In contrast, some studies have found an increasing prevalence of burnout syndrome with the advancement of medical studies [8, 45, 46], while some studies did not [25, 47]. A study in Lebanon [35] found that first-year students were significantly more likely to have burnout syndrome. Our faculty has only recently started enrolling a smaller number of students and at the time of this study the smaller number of students per year was present only until the third academic year, which may be a possible explanation for the differences found. In our study, medical students were enrolled in the faculty in two ways: one group that enrolled while partial "Bologna" was implemented (from fourth to sixth academic year) and the other group that enrolled to studies while complete "Bologna" was implemented (from first to third academic year). Also, our medical students from first to third years of studies attended classes in much smaller groups and had tutors, while students from fourth to sixth years of studies attended classes in much larger groups and without the help of tutors. Some authors attribute this difference to the gradual and better adaptation of older medical students to the new environment, which contributes to a lower risk of burnout [48]. The curriculum, as well as the pedagogical format of teaching at medical faculties, is related to the burnout syndrome [49, 50]. The question remains as to how much the problem of burnout syndrome involves the used learning strategy, in this case the problem-oriented learning method, and how much it involves the very way this strategy is used, especially because systematic literature reviews indicate better knowledge and satisfaction among those students which have been taught by this method [51].

The use of sedatives was recorded in 3.4% of medical students in our study. In a study at the University of Belgrade, the use of sedatives was recorded in 2.6% of medical students and 2.3% of veterinary students, as well as 0.9% of economics students, but without association with burnout syndrome [52]. In a study in Brazil [53], about 12% of medical students of all years of studies used anxiolytics, significantly more female students than male students (15.1% vs. 8.8%, p = 0.038). In a multicenter study in France [54], in the medical student population first-year students consumed 1.5 times more anxiolytics than second-year students: the study authors attributed this result to first-year students’ pressure to pass the first year’s exams, resulting in a higher rate of mood disorders and anxiety. In contrast to first-year students, who spent most of their time preparing for their exam and more often reported study difficulties as motives for psychoactive substances use, second-year students were more likely to look for effects like sedation and stimulation, suggesting greater difficulty in combining studies with social life. According to French authors, medical students have a high rate of substance use and it is unclear whether such behavior is a consequence of self-medication attempts (eg coping with stress) or other motives (including pleasure / novelty seeking) [54]. Among medical students in Cameroon, a significant interaction has been observed between different predictors (chronic disease, alcohol consumption and burnout syndrome) and outcomes—recreational drugs use [55]. In a recent meta-analysis, Koutsimani and co-authors suggest that there is a link between burnout and depression, as well as between burnout and anxiety [56]. The question is whether the use of drugs (antidepressants, sedatives) can be an indicator of the existence of these basic pathologies, or make the appearance of burnout symptoms more probable. A study in India, which included examining medical students at the beginning and at the end of the first year of study, showed a significant increase in depression and stress but not burnout [57]. In the higher years of study, especially among female students, the prevalence of depression and anxiety, as well as psychological distress, is higher compared to the general population [58]. It is uncertain whether the use of sedatives directly leads to higher burnout or whether students who already have poor results and experience high levels of stress turn to recreational drugs use as a source of comfort [55]. As education on drugs abuse, as well as on alcohol and illicit substances use, is part of the curriculum of medical studies, this link should be explored in future study projects.

Some differences between the findings of studies of burnout prevalence and associated factors in medical students can be explained by variations in the observed populations (by gender, age, study year, lifestyle habits, comorbidity) and academic curricula, study design, application of different questionnaires, use of non-validated questionnaires, variations of burnout concept, different response rates. Also, in some studies, different cut-off scores were used to classify burnout using the same questionnaire [16]. Future, primarily longitudinal, studies are needed to explore the link between burnout and certain risk factors in order to establish preventive measures for burnout among medical students.

### Strengths and limitations

To the best of our knowledge, this is one of the few studies dedicated to the identification of predictors for high risk of burnout syndrome in medical students in Serbia, it is one of the few studies that applied the validated Serbian version of MBI-SS, with high the response rate (90.9%). However, this study has several limitations. In addition to the known shortcomings of the cross-sectional study design, the limitation of this study is in the use of self-report questionnaire. Although the principle of anonymity has been applied in the survey, the information bias cannot be ruled out with certainty. Additionally, it is possible that participants sometimes changed their answers in different ways, depending on the environment/neighbours, which can be a source of response bias in this study. Also, this study focused on personal variables rather than environmental factors and did not provide data on other circumstances that could influence the onset of burnout in medical students (such as socioeconomic status, etc). In order to overcome the limitations encountered in this study, further research should be conducted by applying the prospective cohort study design as a more appropriate approach for burnout syndrome assessment in medical students. Firstly, it is particularly needed to identify high risk of burnout syndrome in medical students either before they have entered medical faculty or early in their medical studies enrollment. Finally, the cohort study could provide more detailed continuous psychological evaluation of medical students, direct assessment of potential risk factors for burnout syndrome, with direct insight into the occurrence of burnout syndrome during the entire duration of medical studies.

## Conclusion

This study showed significantly higher prevalence of high risk for burnout syndrome in male than in female medical students. The year of study was significantly associated with increased burnout level in medical students of both genders, while the use of sedatives was associated with high risk for burnout in female medical students.
